# Clinical presentation of acute primary angle closure during the COVID-19 epidemic lockdown

**DOI:** 10.3389/fmed.2022.1078237

**Published:** 2022-12-16

**Authors:** Li Zhou, Shaoqun Wu, Yong Wang, Xianyi Bao, Tingting Peng, Wenjing Luo, Julio Ortega-Usobiaga

**Affiliations:** ^1^Aier Eye Hospital of Wuhan University (Wuhan Aier Eye Hosptial), Wuhan, China; ^2^Department of Cataract and Refractive Surgery, Clínica Baviera, Aier Eye Hospital, Bilbao, Spain

**Keywords:** acute primary angle closure, APAC, glaucoma, COVID-19, lockdown

## Abstract

**Purpose:**

This study aimed to investigate the clinical presentation of acute primary angle closure (APAC) during the COVID-19 epidemic lockdown in Wuhan.

**Methods:**

Consecutive patients seeking APAC treatment at the Wuhan Aier Eye Hospital during the 76 days (January 23–April 8, 2020) when the lockdown policy was implemented due to the COVID-19 pandemic were compared to those during the same period the following year (January 23–April 8, 2021), when the lockdown policy was not implemented. The cohorts were compared to assess demographic variables and clinical presentations.

**Results:**

A total of 54 patients (64 eyes) were included in the 2020, compared with 46 patients (51 eyes) in the 2021. Demographic factors were similar between the groups. Significantly more patients developed blindness in the 2020 cohort (21.87%) than in the 2021 cohort (7.84%). Patients in the 2020 showed a longer time from symptom to treatment (241.84 ± 211.95 h in 2020 vs. 121.53 ± 96.12 h in 2021; *P* = 0.001), higher intraocular pressure at presentation (52.63 ± 12.45 mmHg in 2020 vs. 45.16 ± 9.79 mmHg in 2021; *P* = 0.001), larger pupil diameter (5.47 ± 1.62 mm in 2020 vs. 4.33 ± 1.27 mm in 2021; *P* = 0.001), and more glaucomatous optic neuropathy diagnoses [20/64 eyes (31.25%) in 2020 vs. 7/51 eyes (13.73%) in 2021; *P* = 0.03].

**Conclusion:**

The time between the onset of APAC symptoms and its treatment during the COVID-19 epidemic lockdown was significantly prolonged, which increased the blindness rate of APAC patients.

## Introduction

Acute primary angle closure (APAC) is a type of primary angle-closure glaucoma (PACG) and an important cause of blindness in East Asia (1). In China, it is estimated that PACG afflicts 3.5 million people, and 28 million have narrow anterior chamber angles, which is considered an important risk factor for PACG (2). Although not all PACG patients will have APAC, Asians have a much higher incidence rate of APAC compared to the European population. The crude incidence rates are 10.4 and 3.9 per 100,000 people per year in the above 30-year-old population of Hong Kong and Europe, respectively (3). APAC is accompanied by a rapid increase in intraocular pressure (IOP). If not treated in time, APAC with high IOP can lead to a sharp decline in vision or blindness due to glaucomatous optic neuropathy (GON), which is often accompanied by nausea, vomiting, and other systemic symptoms (4). The management of acute PACG lies in the rapidity of IOP control and the almost instantaneous relief of severe symptoms. Rapid IOP control limits the extent of ocular tissue damage and the resulting ischemia (5).

Since early 2020, the world has faced the mounting challenge of the COVID-19 pandemic. In response to this pandemic, many governments issued national or local lockdowns with the aim of containing the rates of infection and reducing anticipated pressure on hospitals (6). Moreover, additional restrictions, such as strict home isolation, were advised for “clinically vulnerable” (e.g., those aged 70 years or older) and “clinically extremely vulnerable” (e.g., patients on immunosuppression therapies) individuals. Pandemics can influence patient behavior in seeking emergency medical treatment (7). Poyser et al. (8) reported that a 53% reduction in emergency eye department attendance was noted during the lockdown (8). This raises the possibility that some people may have been harmed by not accessing appropriate treatment in a timely manner. It is unclear whether a similar trend was seen among APAC patients. Wuhan, China, began experiencing the outbreak of COVID-19 in January 2020. To control the expansion of the epidemic, the Wuhan government adopted a lockdown measure, which lasted from January 23, 2020 to April 8, 2020. The purpose of this study was to compare the clinical characteristics and treatment prognoses of patients with APAC during and after the COVID-19 epidemic in Wuhan, China.

## Methods

This retrospective study included patients with APAC attending glaucoma clinics at the Aier Eye Hospital of Wuhan University. Consecutive cases of APAC from January 23, 2020 to April 8, 2020 were set as the research group, and consecutive cases of APAC from January 23, 2021 to April 8, 2021 were set as the control group. Detailed diagnosis of APAC was defined by the following criteria, which were modified from previous studies (9): (1) presence of at least two of the following symptoms: ocular or periocular pain, nausea and/or vomiting, and an antecedent history of intermittent blurring of vision with halos; (2) a presenting IOP of > 21 mm Hg; and (3) the presence of at least one of the following signs: conjunctival hyperemia, cornea edema, a mid-dilated unreactive pupil, and a shallow anterior chamber. GON was defined as loss of neuroretinal rim with a vertical cup:disc (C/D) ratio of ≥ 0.6 and/or notching attributable to glaucoma (10). The exclusion criteria of this study were patients presenting with secondary angle closure, such as dislocated lens-induced glaucoma, neovascular glaucoma, uveitic glaucoma, or glaucoma after retinal laser treatment or surgery (11).

All patients with APAC received similar initial treatment: antiglaucomatous eye drops to reduce IOP or anterior chamber paracentesis for rapidly lowering IOP, followed by either yttrium aluminum garnet (YAG) laser peripheral iridotomy (LPI) and/or argon laser peripheral iridoplasty (ALPI) (12). Patients who failed medical and laser therapy underwent lens extraction with or without goniosynechialysis or trabeculectomy. Medical and surgical records prior to and up to 3 months post-treatment of APAC were collected for analysis. For patients with unilateral APAC, the affected eye was selected for data collection. For patients with bilateral APAC, information from both eyes was collected. In this study, all patients were followed up for more than 6 months.

Data include demographics of each patient, time from symptoms to treatment (TST, hours), uncorrected distance visual acuity (UDVA), corrected distance visual acuity (CDVA), IOP, which was measured by an I-care tonometer using a fresh probe for each patient (during the COVID-19 pandemic, the non-contact tonometer was avoided because it can produce aerosols when examining IOP, resulting in virus spread), (13) cornea edema with slit lamp exam [graded from 1 to 3; 1 = no corneal edema; 2 = mild corneal edema (iris details are clear); and 3 = severe corneal edema (iris details obscured)], (14) ocular biometry (IOL Master 700, Zeiss, Jena, Germany), and vertical C/D of the optic nerve. Anterior segment optical coherence tomography (AS-OCT, TOMEY Corp, Aichi, Japan) was used to evaluate the angle. As AS-OCT is a non-contact scan, it was preferred over ultrasound biomicroscopy (UBM), which is a contact scan that can spread infection (15).

### Statistical analysis

All statistical analyses were performed using the Statistical Package for the Social Sciences (SPSS version 20.0; SPSS, Inc.). Descriptive results are presented as the mean and standard deviation for normally distributed continuous variables and as the median and interquartile range for non-normally distributed continuous variables. The *T*-test and Mann–Whitney *U*-test were used to analyze the differences between the two groups for normally distributed and non-normally distributed continuous variables, respectively. For categorical variables, the chi-square test or Fisher's exact test were used to detect differences between the two groups. For all tests, *P*-values < 0.05 were considered statistically significant.

## Results

### Patient characteristics

In total, 54 cases (64 eyes) were recruited for the study group, and 46 cases (51 eyes) were recruited for the control group (Table 1). Compared with the control group, there were no significant differences in eye ratio, gender ratio, or age for the study group. The TST of the control group was significantly lower than that of the study group (*p* < 0.01).

**Table 1:** Baseline demographic and clinical characteristics data.

**Characteristics**	**2020 cohort**	**2021 control**	**Statistical value**	***P*-value**
Cases, *n*	54	46		
**Affected eye**, ***n*** **(%)**	64	51	*X^2^*= 1.14	0.29
Bilateral	10 (18.52%)	5 (10.87%)		
Single	44 (81.48%)	41 (89.13%)		
**Gender**, ***n*** **(%)**			*X^2^* = 0.48	0.49
Male	15 (27.78%)	10 (21.74%)		
Female	39 (72.22%)	36 (78.26%)		
**Age (mean** **±SD), years**	63.19 ± 9.43	65.89 ± 12.11	*t* = −1.25	0.11
**Mean TST, hours (mean** **±SD)**	241.84 ± 211.95	121.53 ± 96.12	*t* = 3.76	0.00

### Clinical characteristics of APAC at presentation

The IOP, pupil diameter (PD), and severe corneal edema (grade 3) in the study group were significantly higher than those in the control group. The CDVA of the study group was worse than that of the control group (Table 2).

**Table 2:** The characteristics of APAC at presentation.

	**2020 cohort**	**2021 control**	**Statistical value**	***P*-value**
Affected eyes, *n*	64	51		
IOP at presentation, mm Hg (mean ± SD)	52.63 ± 12.45	45.16 ± 9.79	*t* = 3.51	0.00
CDVA at presentation (LogMar)	1.53 ± 0.44	1.21 ± 0.39	*t* = 4.07	0.00
PD, mm	6.41 ± 1.73	5.02 ± 1.54	*t* = 4.49	0.00
**Cornea edema at presentation, grade**, ***n*** **(%)**			*X^2^*= 8.04	0.02
Grade 1	2 (3.12%)	4 (7.84%)		
Grade 2	28 (43.75%)	33 (64.71%)		
Grade 3	34 (53.13%)	14 (27.45%)		

### Ocular biometry of APAC

The biometric parameters, including axial length (AL), anterior chamber depth (ACD), and lens thickness (LT), were recorded. There was no significant difference in AL and ACD between the two groups. The LTs in the study group were significantly higher than those in the control group, and the difference was statistically significant (Table 3).

**Table 3:** Biometric parameters of both groups.

**Parameters**	**2020 cohort**	**2021 control**	**Statistical value**	***P*-value**
Affected eyes, *n*	64	51		
AL, mm	22.44 ± 0.84	22.52 ± 0.75	*t* = −0.53	0.29
ACD, mm	1.94 ± 0.48	2.06 ± 0.37	*t* = −1.47	0.07
LT, mm	5.44 ± 1.13	4.96 ± 0.93	*t* = 2.44	0.00

### Treatment methods of APAC

All patients received treatment. The proportion of patients receiving antiglaucoma medications alone and laser treatment (including LPI and/or ALPI) in 2020 was lower than that in 2021, and the proportion of patients receiving surgical treatment (with or without goniosynechialysis) in 2020 was higher than that in 2021. However, there was no significant difference in the proportion of treatment methods between the two groups (Table 4).

**Table 4:** Treatment Methods of APAC.

**Treatment methods**	**2020 cohort**	**2021 control**	**Statistical value**	***P-*value**
Eyes, *n* (%)	64	51	*X^2^* = 1.93	0.38
Medications	4 (6.25%)	4 (7.84%)		
Laser	8 (12.50%)	11 (21.57%)		
Surgery	52 (81.25%)	36 (70.58%)		

### Outcome of APAC treatment

The results after treatment showed that the IOP returned to the normal range in both groups. The postoperative CDVA of the study group was worse than that of the control group, and the proportion of blindness caused by APAC in the study group (21.87%) was higher than that in the control group (7.84%). The postoperative PD after the operation was smaller than preoperatively, but it was still larger than normal pupils, and the PD in the study group was larger than that in the control group. The average C/D of the optic nerve in the study group was higher than that in the control group. The proportion of GON in the study group (31.25%) was higher than that in the control group (13.73%). The degree of postoperative corneal edema was significantly reduced after surgery, but some patients in the study group still maintained moderate corneal edema (Table 5).

**Table 5:** Outcome of APAC treatment.

	**2020 cohort**	**2021 control**	**Statistical value**	***P-*value**
Affected eyes, *n*	64	51		
IOP at follow up (mm Hg); mean ± SD	13.21 ± 3.54	12.79 ± 3.28	*t* = 0.65	0.26
CDVA at follow up (LogMar)	0.48 ± 0.52	0.26 ± 0.21	*t* = 2.84	0.00
Percentage with blindness (≤ Snellen VA 3/60)	21.87% (14/64)	7.84% (4/51)	*X^2^*= 4.23	0.03
PD (mm)	5.47 ± 1.62	4.33 ± 1.27	*t* = 4.12	0.00
C/D at follow up	0.47 ± 0.51	0.38 ± 0.32	*t* = 0.49	0.31
Percentage with GON, % (*n*)	31.25% (20/64)	13.73% (7/51)	*X^2^*= 4.85	0.03
**Cornea edema at follow up, grade**, ***n*** **(%)**			*X^2^*= 2.02	0.15
Grade 1	50 (78.12%)	45 (88.24%)		
Grade 2	14 (21.88%)	6 (11.76%)		
Grade 3	0	0		

## Discussion

PACG is a leading cause of irreversible blindness worldwide, and it predominantly affects Asian populations (16). APAC is a subtype of PACG and an ocular emergency that requires immediate reduction of elevated IOP to prevent permanent vision loss (17). APAC is related to the anatomical factors of the patient's eyeball, especially in the Asian population, including a short AL, a shallow ACD, and a high LT (18). Our results showed that the AL was < 22.5 mm and the depth of the ACD was < 2.5 mm in both groups. Previous studies by Lei et al. (19) showed that nearly 12% of Chinese elderly participants had an ACD < 2.5 mm. Therefore, the prevention and treatment of glaucoma should be strengthened for the elderly population with similar ocular biometry, such as short AL. In addition to biometric parameters, increased tension or stress is a predisposing factor for APAC. Previous studies have evaluated the impact of exam emergency stress on students' IOP (20). The results showed that stress can significantly increase IOP. This could be important when treating glaucoma patients. COVID-19 resulted from a zoonotic virus spread by human-to-human transmission and was declared a pandemic by the World Health Organization on March 11, 2020. Many studies have shown that the COVID-19 pandemic and subsequent lockdown measures led to psychological distress, which may have also increased the incidence of APAC (21).

High IOP in APAC can result in damage to the optic nerve and cause losses in the visual fields while simultaneously altering lens, iris, and corneal endothelial cell (CEC) function (22). The results of our study showed that patients' IOP at presentation in the 2020 group (52.63 ± 12.45 mmHg) was significantly higher than that in the 2021 group (45.16 ± 9.79 mmHg). Our results also showed that the C/D ratio of the study group was higher than that of the control group, and the ratio of GON caused by high IOP in 2020 (31.25%) was higher than that in 2021 (13.73%). There are many mechanisms of optic nerve injury caused by high IOP. The most obvious axonal change that occurs following elevated IOP is the loss of the axons themselves. As presented above, this is most readily apparent by the assessment of optic nerve cross sections, where increased numbers of degenerated axons and reduced axonal counts can be seen (23).

High IOP may cause eye pain, conjunctival hyperemia and other symptoms. However, during the epidemic of COVID-19, it is necessary to identify whether conjunctival hyperemia is caused by glaucoma or COVID-19 (24). High IOP also damages CECs. Li et al. (25) showed that acute ocular hypertension disrupts barrier integrity and pump function in rats' CECs (25). Our study showed that the proportion of severe corneal edema caused by IOP was significantly higher in 2020 than in 2021. Mydriasis is also a common complication of APAC. High IOP can lead to iris ischemia and atrophy, resulting in mydriasis. Li et al. (26) study showed that 62% of patients had iris atrophy within 1 week of APAC attack (26). Mydriasis not only affects vision but also increases complications, such as glare. Our study showed that the pupils of the two groups were significantly dilated and that iris atrophy was difficult to recover from, even after the IOP decreased. Moreover, the degrees of iris atrophy and mydriasis were positively correlated with the duration of high IOP. In this study, the postoperative PD in the 2020 group was about 5.47 mm, which was significantly higher than that in the control group.

The choice of APAC treatment needs to comprehensively evaluate the patient's IOP, degree of corneal edema, degree of angle closure, degree of lens opacity, C/D of the optic nerve, economic ability, and follow-up compliance (27). Rapid reduction of IOP is key to the treatment of APAC. The IOP of APAC is usually > 30 mmHg or even > 50 mmHg. Many studies recommend that APAC treatment should begin with anterior chamber paracentesis to reduce IOP, followed by antiglaucomatous drugs (28). After full evaluation, laser treatment or surgery should be selected. Recent evidence supports initial clear lens extraction in the context of PACG or APAC with an IOP > 30 mmHg (29). In our study, 80% of patients received lens extraction with or without goniosynechialysis.

Unlike other forms of glaucoma, APAC is not only preventable and treatable but can also have excellent outcomes. TST is key to reducing blindness after APAC. Li reported that if a patient with APAC was treated within 4.6 h of the onset of an acute attack, the risk of blindness would be 1% or less (30). Our study showed that CDVA at presentation and postoperative follow-up were worse in the 2020 group than in the 2021 group, and the blindness rate caused by APAC during the epidemic in 2020 (21.87%) was twice that in the same period in 2021 (7.84%). Further analysis of TST data found that the average TST in 2020 was twice that in 2021, because the COVID-19 in 2020 has implemented the lockdown policy. The COVID-19 pandemic strained healthcare resources and led to stay-at-home orders and the closure of non-essential activities, including non-urgent healthcare services (31). In addition to those patients directly infected, the outbreak impacted patients seeking care for medical problems unrelated to COVID-19. One survey showed that among 4,975 U.S. adult respondents, 40.9% reported having delayed or avoided any medical care, including urgent or emergency care (12.0%) and routine care (31.5%), because of concerns about COVID-19 (32). Patel et al. (33) showed that patients with primary rhegmatogenous retinal detachment during the 2020 COVID-19 pandemic were less likely to have macula-on disease, likelier to delay seeking treatment, and they showed worse vision and primary proliferative vitreoretinopathy (33). During the lockdown, although the government had no restrictions on patients going out for medical treatment, the TST of APAC patients was longer, which may be related to patients' fear of COVID-19 or the reduction of transportation and inconvenient travel for elderly patients. Our study found that the number of APAC patients in our hospital in 2020 was higher than that in 2021. This may be due to the higher incidence rate of APAC in 2020. It may also be that during the COVID-19 epidemic lockdown, the medical resources of many general hospitals were used to treat COVID-19 patients, while our hospital specializes in receiving ophthalmic patients and does not see COVID-19 patients. Therefore, more APAC patients may have come to our hospital.

To reduce TST, health education on glaucoma needs to be strengthened. Li et al. (34) reported that while patients' awareness and knowledge levels of glaucoma tend to be limited, they are significantly enhanced after educational intervention. Mobile-based education is essential to ameliorate low public awareness of glaucoma (34). During 2020, Sanjay reported that 46% of ophthalmologists ceased to operate on their patients, and almost 40% were doing < 25% of their original number of surgeries. After consulting doctors by telephone or online, 41% of patients chose to go to the hospital again (35).

Our study showed that the treatment time for APAC during the COVID-19 epidemic lockdown was significantly prolonged, resulting in high IOP, mydriasis, and GON, which increased the blindness rate of APAC. Given the persistence of COVID-19 across the world and the potential for recurrent waves of transmission, the urgent need for continued health education regarding APAC and continued ophthalmologic care is clear.

## Data availability statement

The raw data supporting the conclusions of this article will be made available by the authors, without undue reservation.

## Ethics statement

The studies involving human participants were reviewed and approved by the Institutional Review Board of the Aier Eye Hospital of Wuhan University. The patients/participants provided their written informed consent to participate in this study.

## Author contributions

SW designed the concept of this work and drafted this manuscript. YW generated the overall concept and provided feedback to revise drafts. LZ, XB, and JO-U critically reviewed the manuscript. TP and WL were responsible for identifying and interpreting relevant studies. All authors have read and approved the final manuscript.

## References

[1] Chan EW Li X Tham YC Liao J Wong TY Aung T. Glaucoma in Asia: regional prevalence variations and future projections. Br J Ophthalmol. (2016) 100:78–85. 10.1136/bjophthalmol-2014-306102 26112871

[2] Congdon NG Friedman DS. Angle-closure glaucoma: impact, etiology, diagnosis, and treatment. Curr Opin Opthalmol. (2003) 14:70–3. 10.1097/00055735-200304000-00002 12698044

[3] Chan PP Pang JC Tham CC. Acute primary angle closure-treatment strategies, evidences and economical considerations. Eye. (2019) 33:110–9. 10.1038/s41433-018-0278-x 30467424 PMC6328531

[4] Jonas JB Aung T Bourne RR Bron AM Ritch R Panda-Jonas S. Glaucoma. Lancet. (2017) 390:2183–93. 10.1016/S0140-6736(17)31469-1 28577860

[5] Lam DS Chua JK Tham CC Lai JS. Efficacy and safety of immediate anterior chamber paracentesis in the treatment of acute primary angle-closure glaucoma: a pilot study. Ophthalmology. (2002) 109:64–70. 10.1016/S0161-6420(01)00857-0 11772581

[6] Di Domenico L Pullano G Sabbatini CE Boëlle PY Colizza V. Impact of lockdown on COVID-19 epidemic in Île-de-France and possible exit strategies. BMC Med. (2020) 18:240. 10.1186/s12916-020-01698-4 32727547 PMC7391016

[7] De Filippo O D'Ascenzo F Angelini F. Reduced rate of hospital admissions for ACS during COVID-19 outbreak in Northern Italy. N Engl J Med. (2020) 383:88–9. 10.1056/NEJMc2009166 32343497 PMC7224608

[8] Poyser A Deol SS Osman L Kuht HJ Sivagnanasithiyar T Manrique R. Impact of COVID-19 pandemic and lockdown on eye emergencies. Eur J Ophthalmol. (2021) 31:2894–900. 10.1177/1120672120974944 33213198 PMC8606945

[9] Foster PJ Buhrmann R Quigley HA Johnson GJ. The definition and classification of glaucoma in prevalence surveys. Br J Ophthalmol. (2002) 86:238–42. 10.1136/bjo.86.2.238 11815354 PMC1771026

[10] Ho H Chew PT Sng C Huang H Aung T Perera SA. Comparison of two approaches to managing acute primary angle closure in Asian eyes. Clin Ophthalmol. (2013) 7:1205–10. 10.2147/OPTH.S41674 23818755 PMC3693841

[11] Aung T Friedman DS Chew PT Ang LP Gazzard G Lai YF. Long-term outcomes in Asians after acute primary angle closure. Ophthalmology. (2004) 111:1464–9. 10.1016/j.ophtha.2003.12.061 15288972

[12] Liang YB Wang NL Rong SS Thomas R. Initial treatment for primary angle-closure glaucoma in China. J Glaucoma. (2015) 24:469–73. 10.1097/IJG.0000000000000075 24786102

[13] Tejwani S Angmo D Nayak BK Sharma N Sachdev MS Dada T Sinha R. Preferred practice guidelines for glaucoma management during COVID-19 pandemic. Indian J Ophthalmol. (2020) 68:1277–80. 10.4103/ijo.IJO_1724_20 32587151 PMC7574094

[14] Yang X Su W Wang M Bai Y Li Y Ge J. Effect of anterior chamber paracentesis on initial treatment of acute angle closure. Can J Ophthalmol. (2013) 48:553–8. 10.1016/j.jcjo.2013.04.012 24314422

[15] Ishikawa H. Anterior segment imaging for glaucoma: OCT or UBM? Br J Ophthalmol. (2007) 91:1420–1. 10.1136/bjo.2007.121038 17947263 PMC2095437

[16] Quigley HA Broman AT. The number of people with glaucoma worldwide in 2010 and 2020. Br J Ophthalmol. (2006) 90:262–7. 10.1136/bjo.2005.081224 16488940 PMC1856963

[17] Foster PJ Johnson GJ. Glaucoma in China: how big is the problem? Br J Ophthalmol. (2001) 85:1277–82. 10.1136/bjo.85.11.1277 11673287 PMC1723754

[18] Tarongoy P Ho CL Walton DS. Angle-closure glaucoma: the role of the lens in the pathogenesis, prevention, and treatment. Surv Ophthalmol. (2009) 54:211–25. 10.1016/j.survophthal.2008.12.002 19298900

[19] Lei Q Tu H Feng X Ortega-Usobiaga J Cao D Wang Y. Distribution of ocular biometric parameters and optimal model of anterior chamber depth regression in 28,709 adult cataract patients in China using swept-source optical biometry. BMC Ophthalmol. (2021) 21:178. 10.1186/s12886-021-01932-4 33849464 PMC8045194

[20] Jiménez R Vera J. Effect of examination stress on intraocular pressure in university students. Appl Ergon. (2018) 67:252–8. 10.1016/j.apergo.2017.10.010 29122197

[21] Violant-Holz V Gallego-Jiménez MG González-González CS Muñoz-Violant S Rodríguez MJ Sansano-Nadal O. Psychological health and physical activity levels during the COVID-19 pandemic: a systematic review. Int J Environ Res Public Health. (2020) 17:9419. 10.3390/ijerph17249419 33334073 PMC7765528

[22] Wright C Tawfik MA Waisbourd M Katz LJ. Primary angle-closure glaucoma: an update. Acta Ophthalmol. (2016) 94:217–25. 10.1111/aos.12784 26119516

[23] Morrison JC Johnson EC Cepurna W Jia L. Understanding mechanisms of pressure-induced optic nerve damage. Prog Retin Eye Res. (2005) 24:217–40. 10.1016/j.preteyeres.2004.08.003 15610974

[24] Meduri A Oliverio GW Mancuso G Giuffrida A Guarneri C Venanzi Rullo E. Ocular surface manifestation of COVID-19 and tear film analysis. Sci Rep. (2020) 10:20178. 10.1038/s41598-020-77194-9 33214658 PMC7677531

[25] Li X Zhang Z Ye L Meng J Zhao Z Liu Z. Acute ocular hypertension disrupts barrier integrity and pump function in rat corneal endothelial cells. Sci Rep. (2017) 7:6951. 10.1038/s41598-017-07534-9 28761172 PMC5537405

[26] Loon SC Chew PT Oen FT Chan YH Wong HT Seah SK. Iris ischaemic changes and visual outcome after acute primary angle closure. Clin Exp Ophthalmol. (2005) 33:473–7. 10.1111/j.1442-9071.2005.01064.x 16181271

[27] Thomas R Sekhar GC Kumar RS. Glaucoma management in developing countries: medical, laser, and surgical options for glaucoma management in countries with limited resources. Curr Opin Ophthalmol. (2004) 15:127–31. 10.1097/00055735-200404000-00012 15021224

[28] Lu DW Tai MC Chang YH Liang CM Chen CL Chien KH. Anterior chamber paracentesis and pH values in patients with acute primary angle closure. Graefes Arch Clin Exp Ophthalmol. (2013) 251:1229–34. 10.1007/s00417-012-2198-y 23142993

[29] Napier ML Azuara-Blanco A. Changing patterns in treatment of angle closure glaucoma. Curr Opin Ophthalmol. (2018) 29:130–4. 10.1097/ICU.0000000000000453 29194069

[30] Li S Tang G Fan SJ Zhai G Lv J Zhang H. Factors associated with blindness three months following treatment for acute primary angle glaucoma. Br J Ophthalmol. (2021) 105:502–6. 10.1136/bjophthalmol-2020-316259 32769077

[31] Omer SB Malani P Del Rio C. The COVID-19 pandemic in the US: a clinical update. JAMA. (2020) 323:1767–8. 10.1001/jama.2020.5788 32250388

[32] Czeisler MÉ Marynak K Clarke KEN Salah Z Shakya I Thierry JM. Delay or avoidance of medical care because of COVID-19-related concerns—United States, June 2020. MMWR Morb Mortal Wkly Rep. (2020) 69:1250–7. 10.15585/mmwr.mm6936a4 32915166 PMC7499838

[33] Patel LG Peck T Starr MR Ammar MJ Khan MA Yonekawa Y. Clinical presentation of rhegmatogenous retinal detachment during the COVID-19 Pandemic: a historical cohort study. Ophthalmology. (2021) 128:686–92. 10.1016/j.ophtha.2020.10.009 33058938 PMC7550253

[34] Li J Huang W Gao J Li D Xu L Huang J. Impact of mobile-based health education on the awareness and knowledge of glaucoma in Chinese patients. Telemed J E Health. (2019) 25:455–61. 10.1089/tmj.2018.0123 30044202

[35] Sanjay S Leo SW Au Eong KG Adriono GA Fong KC Anand K. Global ophthalmology practice patterns during COVID-19 pandemic and lockdown. Ophthalmic Epidemiol. (2021) 23:1–12. 10.1080/09286586.2021.1934037 34167454

